# A busca pela restituição da importância da historiografia da ciência

**DOI:** 10.1590/S0104-59702025000100004

**Published:** 2025-02-14

**Authors:** Fernanda Schiavo Nogueira

**Affiliations:** i Doutoranda, Programa de Pós-graduação em História/Universidade Federal de Minas Gerais. Belo Horizonte – MG – Brasil. fernandaschiavonogueira@gmail.com

Magistral! Eis a melhor definição para o livro *Handbook for the historiography of science*, organizado por Mauro Condé e Marlon Salomon, lançado em setembro de 2023 pela Springer. O livro representa trabalho de referência para especialidade tão subestimada na atualidade, a historiografia da ciência. Campo do conhecimento que seria caracterizado pela atuação isolada e dispersa de investigadores que publicariam obras pontuais, quase sempre classificadas restritivamente como filosofia da ciência. Apenas tentativas muito localizadas visariam permitir o intercâmbio dos resultados apurados por pesquisas individuais, como congressos eventuais e a publicação dos trabalhos dos participantes. Assim, a obra de Condé e Salomon (2023) constitui espaço de diálogo privilegiado que possibilita a efetiva integração dos profissionais da historiografia da ciência, o que contribui para a consolidação da comunidade de especialistas da área e dos demais interessados.

*Handbook for the historiography of science* foi dividido em quatro eixos temáticos: (1) autores-chave para a historiografia da ciência; (2) conceitos e concepções da historiografia da ciência; (3) historiografia da ciência, da ciência moderna ao mundo científico contemporâneo; (4) historiografia da ciência e campos afins. O livro apresenta como objetivo fornecer as primeiras orientações para quem pretende ingressar na história da ciência e conhecer a historiografia pertinente a esse campo de conhecimento tendo o entendimento das principais discussões estabelecidas dentro da especialidade. *Handbook for the historiography of science* beneficiará estudiosos com graus de capacitação diversos, desde os pesquisadores novatos aos mais experientes.

Os principiantes serão bem guiados na compreensão dos objetos de estudo e das abordagens trabalhadas, fundamentais para a criação de olhar rico e diversificado sobre os temas das investigações do gênero. Aqueles com maior repertório poderão ler integralmente a obra ou consultar os assuntos de interesse para a atualização dos estudos ou para estabelecer interlocução qualificada com os capítulos lidos e utilizar as ideias apropriadas em suas pesquisas. Os dois tipos de investigadores tirarão grande proveito da obra e desfrutarão de plenas condições de utilizar o livro como base para subsidiar o desenvolvimento de contribuições originais e significativas em historiografia da ciência.

*Handbook for the historiography of science*, como indica o próprio título, desempenha como principal função o papel de manual, ou seja, a obra visa à disseminação das ideias essenciais responsáveis pela caracterização de cada assunto discutido. Todavia, o livro ultrapassaria as expectativas do leitor, segundo as quais a leitura seria a mera reprodução da interpretação tradicional dos objetos de estudo. Quase a totalidade dos capítulos conjuga a abordagem de temas canônicos com a introdução de perspectivas novas sobre os assuntos trabalhados. Por exemplo, o capítulo cinco discute as limitações de artigos e livros sobre *Gênese e desenvolvimento de um fato científico* por trabalharem pouco a influência decisiva da biologia como modelo de epistemologia em Ludwik Fleck. O olhar inovador para o tema central adotado ocorre de maneira semelhante em outra parte da obra. O capítulo 15 desafia a visão convencional na qual predomina a interpretação da epistemologia de tradição francesa como opositora ao positivismo predominante no século XIX, e enfatiza a importância de Comte para Bachelard, Canguilhem e Foucault.

O primeiro eixo temático do livro analisa as contribuições dadas à historiografia da ciência por nomes significativos da especialidade. O foco da seção reside nas principais características do pensamento defendido pelos livros e artigos selecionados, como: os assuntos discutidos, os tipos de interpretação trabalhados e outras abordagens referenciadas pelas obras no período em questão. As ideias foram retratadas contextualmente pela inserção das publicações na carreira acadêmica dos autores, pela análise das mudanças da conjuntura histórica na qual viveram e do panorama intelectual da época com a qual dialogavam. O segundo eixo temático avalia os usos de conceitos representativos para a historiografia da ciência em diferentes narrativas da especialidade e debate quais os aspectos essenciais das ideias veiculadas. Apesar de a maior parte da seção trabalhar várias concepções de importância fundamental, dois conceitos centrais são abordados: revolução científica e epistemologia histórica.

O terceiro eixo temático analisa a história da historiografia da ciência. Cada capítulo mapeia os traços gerais apresentados pela estrutura historiográfica assumida pelas narrativas apresentadas por diferentes tipos de história da ciência que emergiram na modernidade. A periodização escolhida na seção abrange desde os séculos XV e XVI, época da emergência da ciência moderna, à renovação da historiografia com a ascensão da antropologia e dos *science studies*, no século XXI. Por fim, o quarto eixo apresenta finalidade dupla. Três dos capítulos da seção desenvolvem a complexa relação estabelecida entre historiografia da ciência e diferentes formas de conhecimento, representadas por religião, história e filosofia. Os outros dois capítulos mostram os desafios enfrentados pela especialidade devido às novas interpretações feitas sobre gênero e relações metrópole/colônia e como a área sofre influência das abordagens atuais.

Poucas são as possíveis lacunas encontradas no livro. São dignos de destaque a não abordagem de autores fundamentais para a historiografia da ciência (como Foucault, Bloor e Latour) e o reduzido número de conceitos importantes utilizados na historiografia da ciência trabalhados ao longo da obra. Todavia, as ausências identificadas no livro não foram ocasionadas pela falha dos organizadores da obra, mas pela limitação de espaço ou pela ausência de especialistas nesses assuntos. Os autores e os conceitos omitidos, embora não sejam o mote de nenhum dos artigos compilados no livro, aparecem em relativo lugar de destaque em outros momentos da obra. Portanto, as aparentes faltas constatadas permitem apenas diagnosticar o quanto seria bem-vinda a continuidade do projeto da Springer com a publicação de um segundo volume do livro em questão.

Publicações como *Handbook for the historiography of science* constituem espaço de discussão vital para a historiografia da ciência. A escolha de interlocutores de qualidade e as instigantes ideias debatidas são intencionalmente projetadas no livro como o convite mais contundente ao desenvolvimento de pesquisas em novas direções dentro da especialidade. Para Michael Dietrich (2023), no prefácio da série, a maioria dos campos de estudo abarcados pela história apresenta extensa literatura dedicada à historiografia. A história da ciência tem, infelizmente, a menor quantidade de recursos quando comparada às demais subáreas da história. Predomina a ideia segundo a qual a história constitui conhecimento dotado de legitimidade exclusivamente na e pela pesquisa de arquivo. Segundo essa concepção, o trabalho de todo e qualquer historiador seria reduzido à pura execução da prática. Assim, a autorreflexão da história, proporcionada pela historiografia, ocuparia posição não suficientemente valorizada.

No limite, a importância exacerbada dada ao trabalho com as fontes conduziria aos perigos do retorno da epistemologia empirista. O historiador, servindo-se dos dados apurados nos documentos, supostamente disponibilizaria os tijolos para a construção de representações exatas do passado, nas quais os “fatos falariam por si”. As discussões estabelecidas na historiografia seriam vistas como tempo desperdiçado, atividade que distanciaria o profissional do que realmente interessa: a pesquisa de arquivo. Contudo, as informações coletadas nas fontes consistiriam em evidências capazes de retratar o vivido exclusivamente quando formuladas teoricamente, segundo as perguntas feitas pelo pesquisador e as respostas obtidas. Portanto, são todos os instrumentos facultados pela historiografia que possibilitam ao historiador melhor preparo intelectual para enfrentar todas as operações fundamentais envolvidas na sua rotina de trabalho profissional. Assim, abordagens historiográficas, como aquelas encontradas em *Handbook for the historiography of science*, tornariam o sujeito do conhecimento menos ingênuo e mais arguto, dotado de maior capacitação para selecionar fontes, criar e direcionar problemas aos documentos escolhidos, elaborar todo o material abordado e alcançar resultados consistentes.
